# Use of a Targeted Exome Next-Generation Sequencing Panel Offers Therapeutic Opportunity and Clinical Benefit in a Subset of Patients With Advanced Cancers

**DOI:** 10.1200/PO.18.00213

**Published:** 2019-03-08

**Authors:** Scott Kopetz, Kenna R. Mills Shaw, J. Jack Lee, Jiexin Zhang, Beate Litzenburger, Vijaykumar Holla, Walter Kinyua, Emily Broaddus, Molly S. Daniels, Funda Meric-Bernstam, Russell R. Broaddus

**Affiliations:** ^1^ University of Texas MD Anderson Cancer Center, Houston, TX

## Abstract

**PURPOSE:**

Smaller hotspot-based next-generation sequencing (NGS) panels have emerged to support standard of care therapy for patients with cancer. When standard treatments fail, it is unknown whether additional testing using an expanded panel of genes provides any benefit. The purpose of this study was to determine if larger sequencing panels that capture additional actionable genes, coupled with decision support, translates into treatment with matched therapy after frontline therapy has failed.

**PATIENTS AND METHODS:**

A prospective protocol accrued 521 patients with a wide variety of refractory cancers. NGS testing using a 46- or 50-gene hotspot assay, then a 409-gene whole-exome assay, was sequentially performed in a Clinical Laboratory Improvement Amendments–certified clinical laboratory. A decision-support team annotated somatic alterations in clinically actionable genes for function and facilitated therapeutic matching. Survival and the impact of matched therapy use were determined by Kaplan-Meier estimate, log-rank test, and Cox proportional hazards regression.

**RESULTS:**

The larger NGS panel identified at least one alteration in an actionable gene not previously identified in the smaller sequencing panel in 214 (41%) of 521 of enrolled patients. After the application of decision support, 41% of the alterations in actionable genes were considered to affect the function of the gene and were deemed actionable. Forty patients (40 of 214 [19%]) were subsequently treated with matched therapy. Treatment with matched therapy was associated with significantly improved overall survival compared with treatment with nonmatched therapy (*P* = .017).

**CONCLUSION:**

Combining decision support with larger NGS panels that incorporate genes beyond those recommended in current treatment guidelines helped to identify patients who were eligible for matched therapy while improving overall treatment selection and survival. This survival benefit was restricted to a small subset of patients.

## INTRODUCTION

Targeted sequencing of individual genes to guide therapy has become the standard of care for several different cancer types, including colorectal cancer *(KRAS*, *BRAF*, and *NRAS*), lung cancer (*KRAS* and *EGFR*), melanoma (*BRAF*), GI stromal tumor (*KIT*), and gliomas (*IDH1* and *IDH2*). With current clinical molecular diagnostics, sequencing is faster, uses less tumor DNA, and costs less when genes are multiplexed into small next-generation sequencing (NGS) panels. Historically, the focus was often on the identification of mutations found in hotspots—areas of the genome where known drivers are frequently mutated, many of which can be mapped to therapeutic intervention. These panels typically emphasize genes that are recommended by cancer treatment guidelines.

An emerging utility of NGS is the screening of other types of cancers for mutations in clinically actionable genes that can be targeted therapeutically. Such treatment approaches are associated with improved survival in retrospective analyses screening,^1^ prospective trials,^2-4^ and meta-analyses,^5-7^ but this approach is not universally embraced.^8,9^ An important issue in these studies is the low number of patients who benefit from this approach. Reasons for this include the low incidence of actionable alterations, ineffective drugs for some targets, limited number of open slots in trials, and an unwillingness to enroll in a trial. Limited access to targeted therapy trials is an issue, as some trials enroll only certain tumor types or are only active in a few geographic sites.

**CONTEXT**

**Key Objective**

Can additional actionable information be identified by expanding sequencing coverage to the full exome of hundreds of genes beyond hotspot analysis and, secondarily, does that additional information affect patient outcomes?

**Knowledge Generated**

Only a small number of genes have alterations that are specifically listed in a US Food and Drug Administration–approved indication that would match a patient with cancer to a specific actionable agent—for example, *BRAF* V600E to vemurafenib. However, we demonstrate that a subset of patients can potentially benefit through expanded panel testing that covers the majority of the actionable-treatment space in oncology. We demonstrate that patients have improved overall outcome when matched to therapies on the basis of molecular alterations present in their tumors compared with similar patients who were not matched.

**Relevance**

A subset of patients that have experienced progression on standard-of-care treatment options could potentially benefit from expanded molecular testing to identify therapeutic options available via clinical trials.

In patients with advanced cancer that is refractory to standard treatment, any benefit gained from the detection of mutations in clinically actionable genes beyond those detected using focused hotspot sequencing panels remains unknown. Hotspot-focused NGS panels fail to robustly identify copy number alterations or mutations that are found outside commonly altered loci, which would represent an advantage of larger NGS panels. The goal of our institutional initiative was to systematically determine the nature of the novel alterations identified in patients for which we previously had sequencing data from a small sequencing panel. We sought to determine if those alterations could be used to place additional patients into matched targeted therapy treatment regimens.

## PATIENTS AND METHODS

### Patient Selection

Patients were prospectively enrolled in an institutional review board–approved institutional protocol (PA14-0099) between May 7, 2014, and October 5, 2015. Enrollment criteria included the following: adult patient with pathologic documentation of a single solid tumor malignancy; completed frontline treatment and any standard treatments that extended life by at least 3 months; Eastern Cooperative Oncology Group performance status of 0 or 1; no active brain metastases; and completed tumor testing using a smaller sequencing panel and either disease progression on a matched therapy that targeted a previously identified actionable finding or no clinically actionable mutations detected.

### Molecular Assays and Decision Support

**FIG A1.**
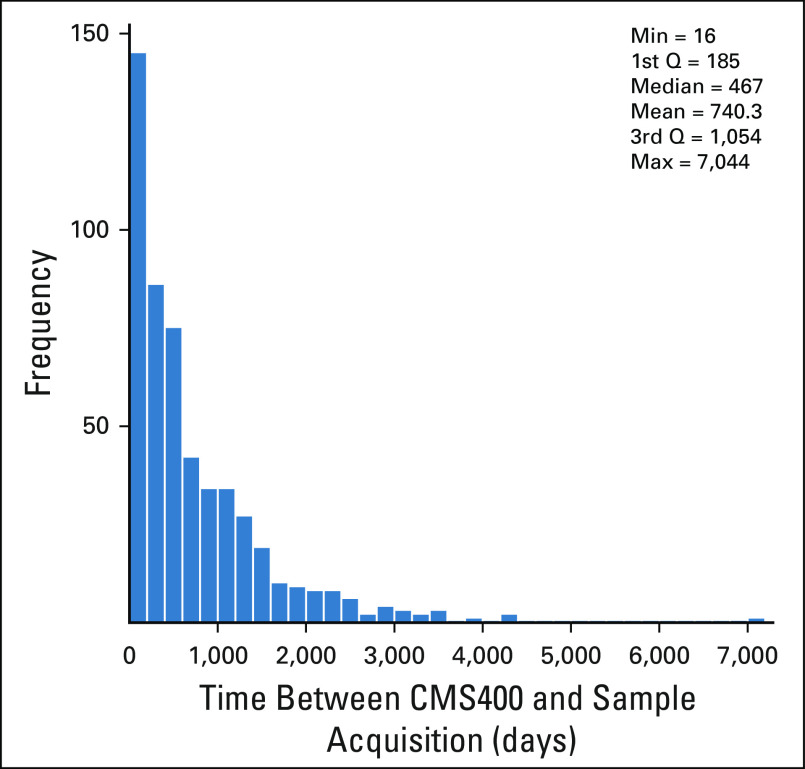
Summary of time from tumor tissue acquisition (surgery or biopsy) to NGS testing. Q, quartile.

Hotspot mutation testing was primarily performed on archival formalin-fixed, paraffin-embedded primary and metastatic cancers acquired during the course of routine clinical care using a 46- or 50-gene NGS panel described previously^10^ (actionable genes summarized in the Data Supplement). Subsequent testing used a larger NGS panel that consisted of the entire coding regions of 409 cancer-related genes with methodologies previously detailed^11^ (actionable genes and details of larger NGS panel provided in the Data Supplement). The protocol did not provide for image-guided biopsies of the most recent metastasis for testing; median time since tissue acquisition to NGS was 467 days (Appendix Fig A1). Relevant germline findings were reported to the referring oncologist.^12^ For comparisons with protocol patients, The Cancer Genome Atlas (TCGA) data—gene mutations and gene amplifications—for the same 409 genes from 316 ovarian high-grade serous carcinomas, 212 colorectal adenocarcinomas, and 230 lung adenocarcinomas were extracted using cBioPortal.org. For patients with colorectal cancer, mismatch repair was assessed as part of routine clinical care.^13^

Somatic mutations and amplifications that were reported for each patient were reviewed by the Precision Oncology Decision Support Team.^14,15^ This involved annotation for function and potential clinical trial matching for actionability. Actionability was limited to the following: genes for which a drug is available that directly or indirectly targets proteins and/or signaling pathways activated as a result of alterations in the gene, and whether there is preclinical or clinical evidence that demonstrates that genomic alterations in the gene are drivers of tumorigenesis and/or confer sensitivity to targeted agents. Somatic alterations in actionable genes were classified for their functional and therapeutic significance using published literature, our internal functional genomics platform,^16,17^ and interpretations of the impact of the mutation on the basis of location within the protein structure. Variants were annotated as actionable, not actionable, or of unknown actionability (Data Supplement). For the smaller 46- and 50-gene NGS panel, 34 and 38 genes, respectively, were considered actionable, whereas for the larger 409-gene NGS panel, 96 genes were actionable (Data Supplement).

### Statistical Analysis

Study sample size was dictated by the capacity of the Clinical Laboratory Improvement Amendments–certified clinical molecular diagnostics laboratory that performed the 409-gene NGS panel, estimated to be 25 cases per week. With a target of 600 patients, it was determined a priori that the rate of identification of alterations in actionable genes could be estimated with a standard error of no more than 0.02. We used Fisher’s exact test to assess whether the gene mutation frequency was significantly different between protocol patients with colorectal adenocarcinoma, lung adenocarcinoma, or ovarian high-grade serous carcinoma and corresponding TCGA data sets. The probability of overall survival was determined by Kaplan-Meier estimate, and we used a log-rank test to assess the statistical significance of the difference between patient groups. To evaluate the effect of clinical variables on overall survival, we performed multivariable survival analysis using a Cox proportional hazards regression model that included variables that were significant (*P* < .05) in a univariable analysis.

## RESULTS

### Characteristics of the Patient Population Enrolled in the Protocol

**FIG A2.**
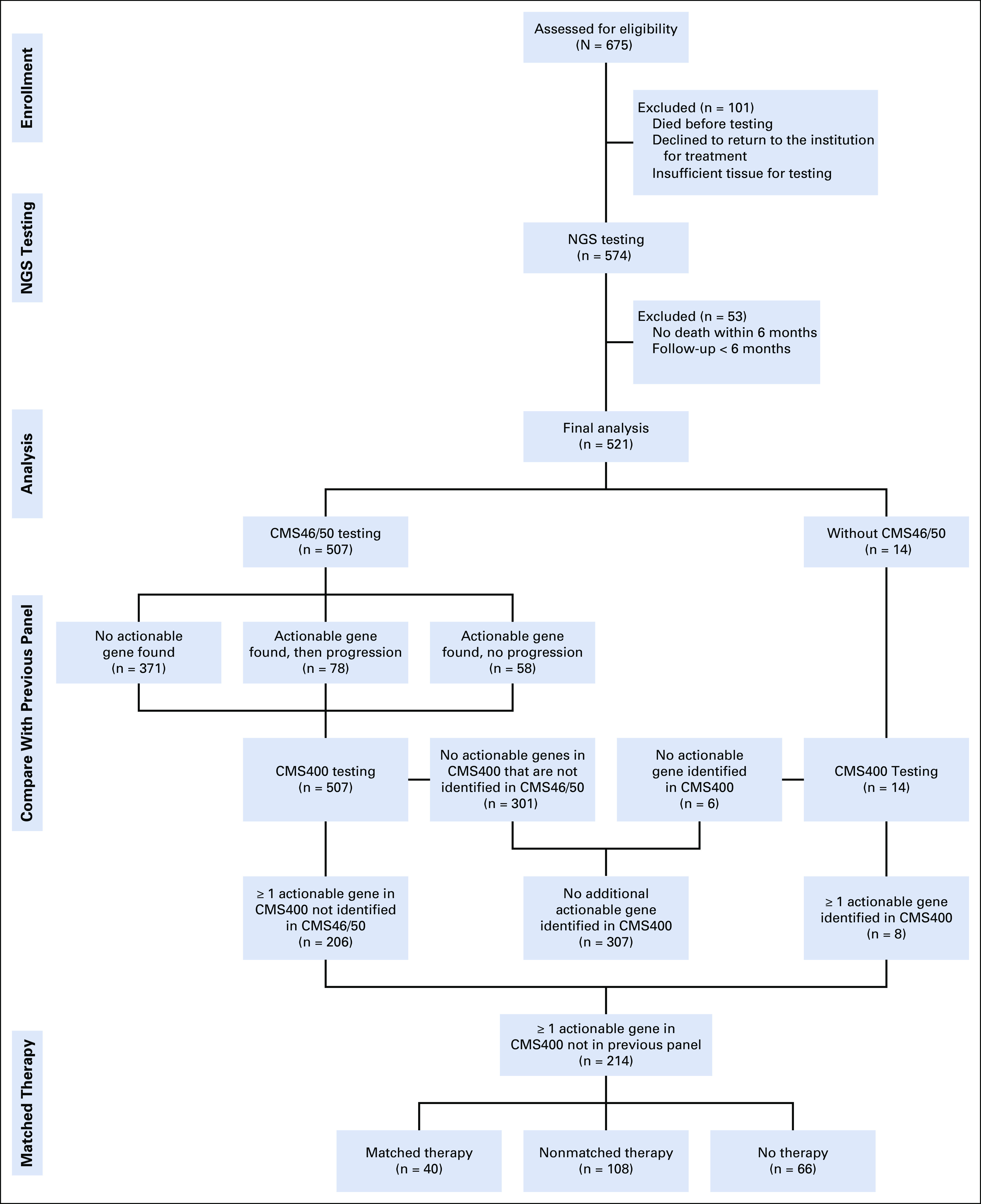
CONSORT diagram summarizing the progress of patients enrolled in the next-generation sequencing (NGS) protocol.

A total of 675 patients were enrolled, with large-panel testing completed in 569 patients. Patient death, declination to return for treatment, and insufficient tissue were the primary reasons for not completing the 409-gene panel NGS testing. Five hundred twenty-one patients who received at least 6 months of follow-up or who died within 6 months were included in the analysis (Appendix Fig A2). Colorectal cancer, sarcoma, head and neck cancer, ovarian cancer, and breast cancer were the most frequently represented cancers in the cohort (Table 1), but the protocol enrolled patients with a broad spectrum of cancer types.

**TABLE 1.**
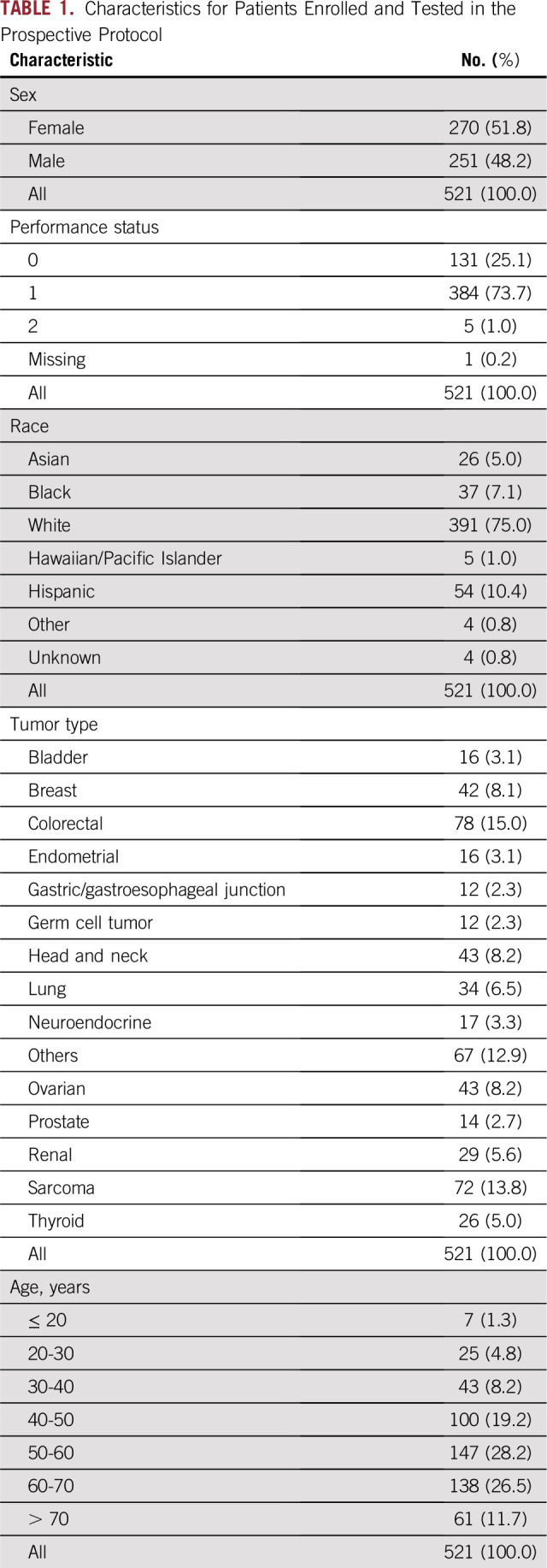
Characteristics for Patients Enrolled and Tested in the Prospective Protocol

### Sequencing Results

**FIG 1.**
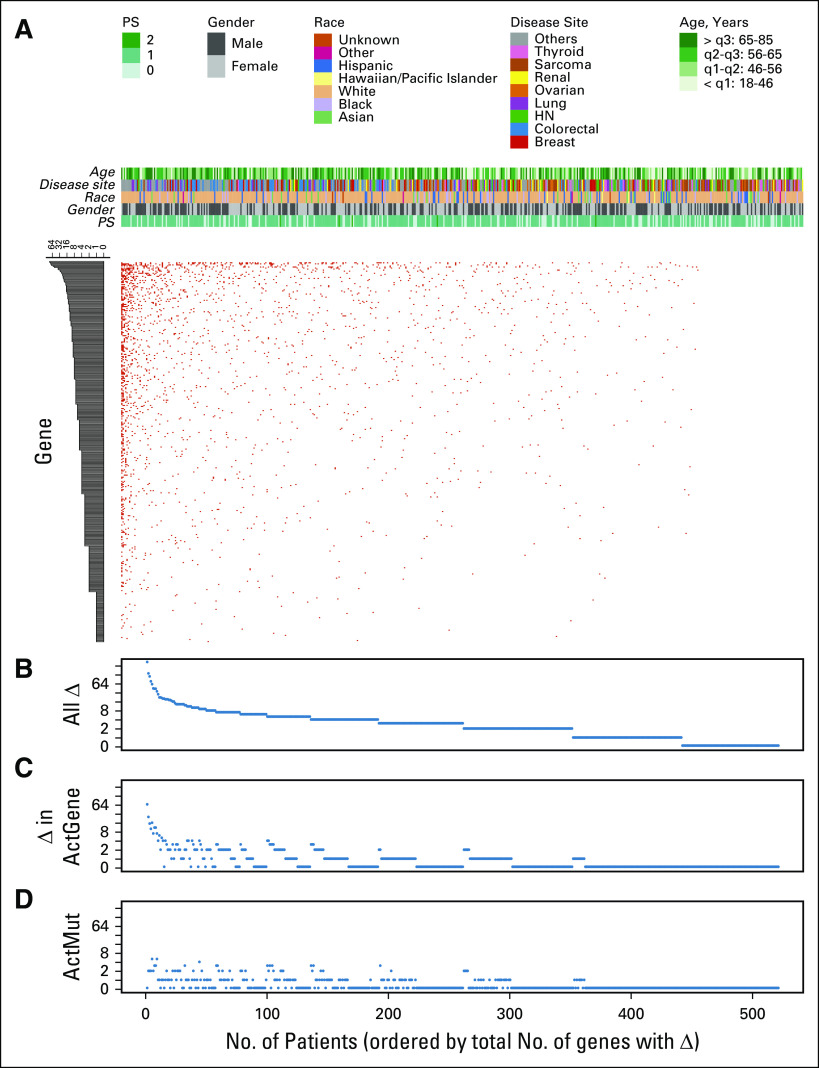
Summary of tumor mutation burden in the protocol patient population after excluding patients who had alterations in actionable genes detected in the smaller sequencing panel. (A) Overall distribution of tumor alterations, with patients arranged horizontally and genes vertically. (B) Total number of gene alterations per tumor ranged from zero to more than 250, with a mean of 5.1 alterations per tumor. (C) Only 96 of 409 genes on the next-generation sequencing panel were considered clinically actionable. On average, only one alteration in an actionable gene per tumor was detected. (D) On average, only 0.4 actionable alterations—mutation or amplification in an actionable gene that is known or has inferred biologic function—were detected per tumor. The majority of alterations in actionable genes identified were variants of unknown significance. HN, head and neck; PS, performance status; q, quartile.

**FIG 2.**
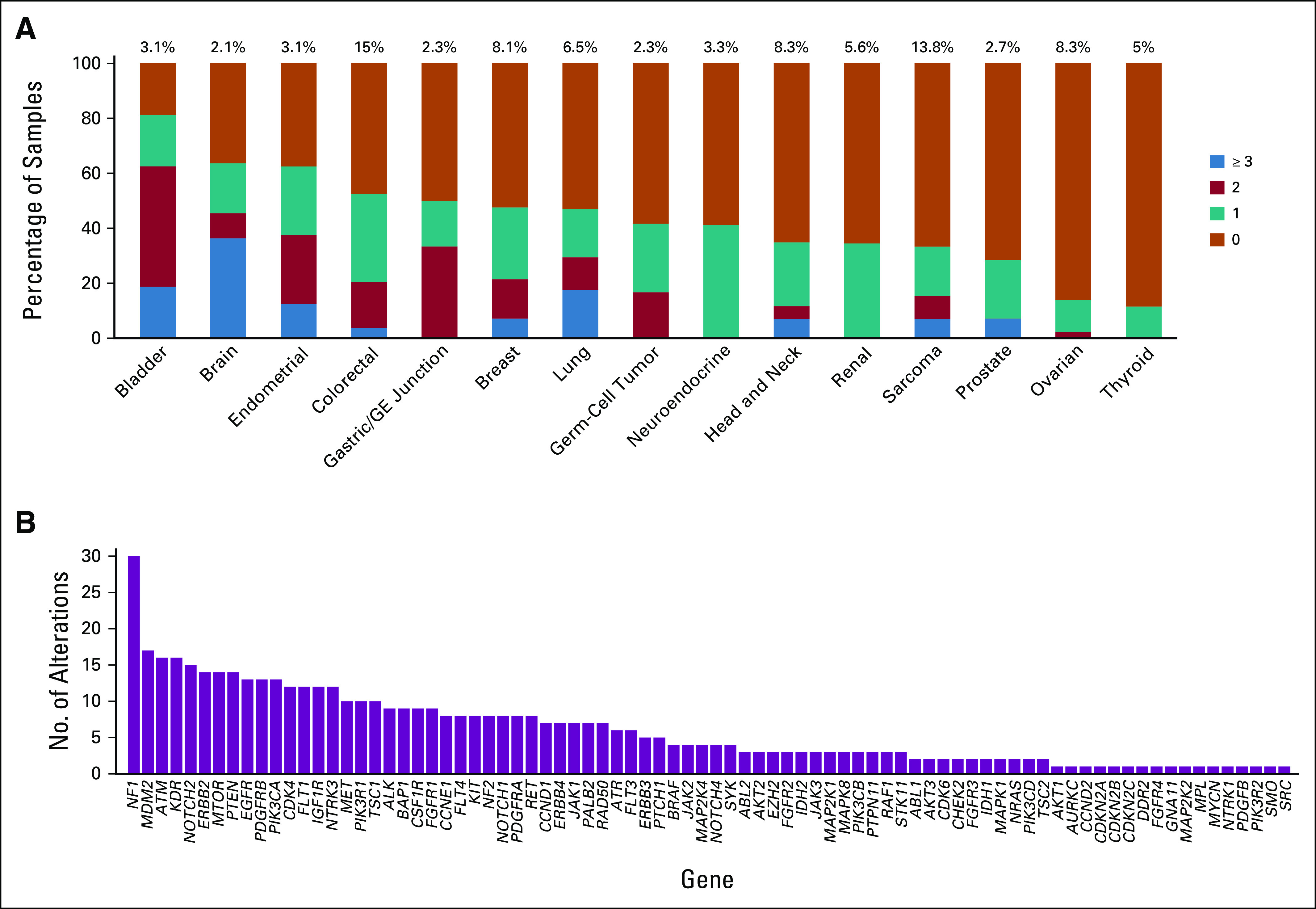
(A and B) Summary of alterations per tumor type (A) and specific genes in which alterations were identified (B). (A) Stacked bar chart shows the percent of cases for each broad class of tumor type listed with 0, 1, 2, or 3 or more alterations in actionable genes not detected in the smaller testing panel. Data are shown for tumor types in which at least 10 patient tumors were sequenced. Total alterations include both mutations and gene amplifications. (B) Alterations were found across a broad spectrum of potentially actionable genes. GE, gastroesophageal.

**FIG A3.**
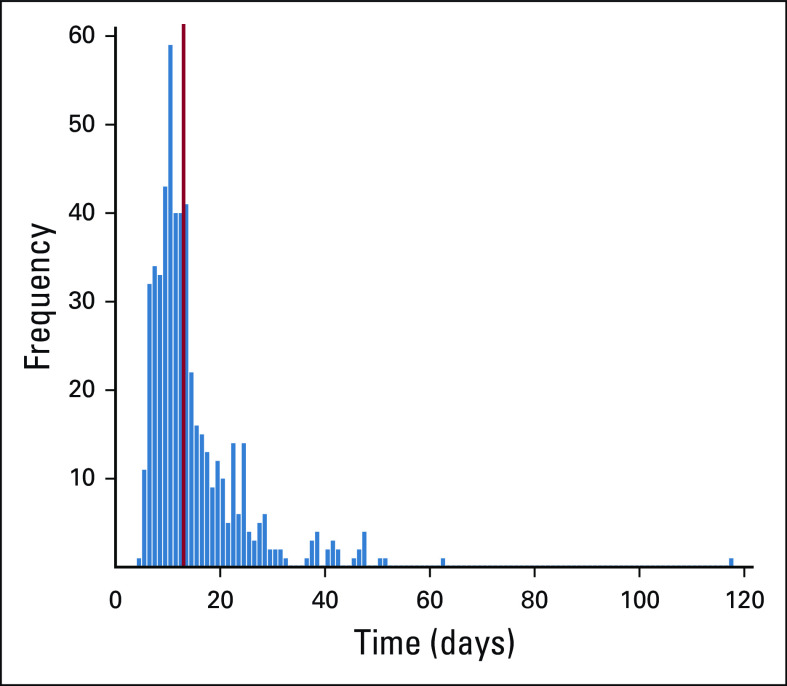
Summary of the turnaround time (in days) for large panel next-generation sequencing testing to be finalized once tissue was received by the clinical molecular diagnostics lab. Red line indicates median (13 days).

Once material was received by the molecular diagnostics laboratory, a median of 13 days passed before the molecular reports were finalized and entered into the electronic medical record (Appendix Fig A3). Of the 521 patients, 71% entered the study without any mutations in actionable genes identified in the smaller NGS panel that would allow for enrollment in a matched targeted therapy clinical trial. Of the 521 patients who were tested with the 409-gene NGS assay, 214 (41%) had at least one tumor alteration in an actionable gene that was not identified in a prior assay using a smaller NGS panel (Figs 1A and 1B). Full 409-gene NGS assay results are summarized for all patients in the Data Supplement. Ten percent of these patients had amplifications in actionable genes that were not detectable in the smaller NGS panels. Not counting the sequencing results from the smaller panel, the mean number of alterations detected in actionable genes in the 409-gene panel was 1.0 (range, 0 to 66; Fig 1C). After the application of decision support, 201 (41%) of 495 alterations in actionable genes were considered to affect the function of the gene and were deemed actionable (mean per patient, 0.4; range, 0 to 5; Fig 1D). The rate of detection of an alteration in an actionable gene with additional testing was dependent on tumor type, with low rates observed in thyroid and ovarian cancers (Fig 2A). Alterations in a variety of actionable genes from the 409-gene panel were identified, with the most common mutations in genes *NF1*, *MDM2*, *ATM*, *KDR*, *NOTCH2, ERBB2*, *MTOR*, and *PTEN* (Fig 2B).

### Matching Tested Patients to Targeted Therapy

Of the 214 patients who had alterations in actionable genes in the large NGS platform that were not detected in the previous small-panel NGS testing, 40 (19%) were matched to a targeted therapy on the basis of their molecular profile within 6 months of receiving test results (Data Supplement). A total of 108 patients in the cohort were treated with unmatched therapy after the completion of large NGS panel testing. These patients had mutations in actionable genes that were classified as actionable, potentially actionable, or a variant of unknown significance but the oncologist chose a therapy that was not a match to the gene alterations found. Reasons for not treating with matched therapy protocols were similar to those from a previously reported patient population^18^ and included declining performance status, a desire to seek treatment closer to home, or an oncologist who did not consider the gene mutation clinically actionable or the targeted therapy sufficiently active in the given tumor type (Data Supplement).

### Decision Support

**FIG 3.**
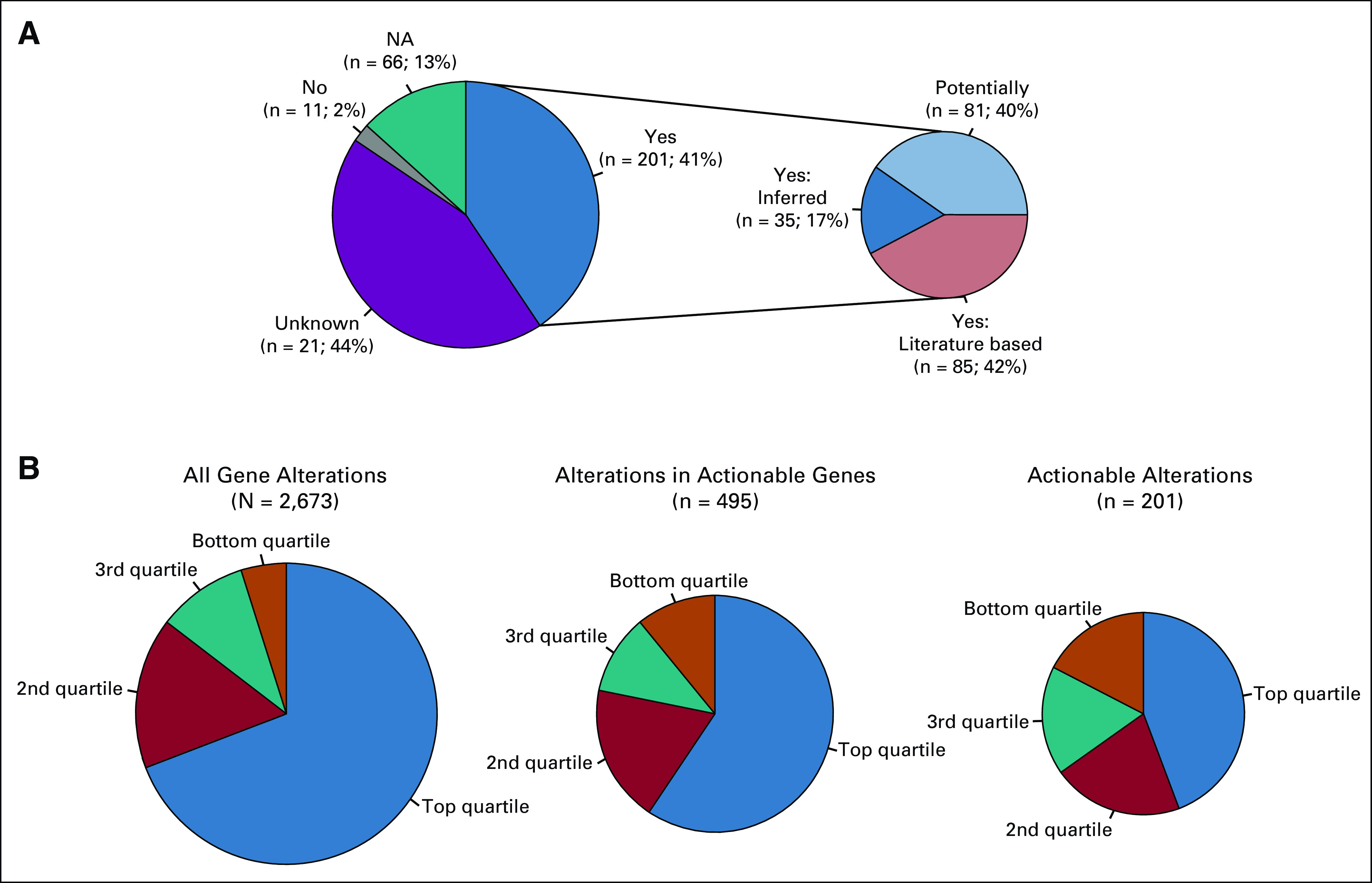
(A) Distribution of variants in actionable genes. Forty-four percent of the alterations found in actionable genes were variants of unknown significance (Unknown), and 13% were not annotated because they were all from one hypermutated patient (NA). Of the alterations with known function, 2% are known to be benign or not actionable (No), with the remainder having some annotated function on the basis of the existing literature. Functional significance was assessed on the basis of the specific variant having a reported function in the literature on the basis of the rules described in Patients and Methods. (B) Distribution of total alterations, alterations in actionable genes, and actionable alterations as organized by quartiles of patients by total mutation load. Presence of clinically actionable alterations was not related to high overall tumor mutation burden.

Decision support was critical as alterations were detected in a number of actionable genes that were less familiar to oncologists, and the availability of matching clinical trials varied during the study period. As previously mentioned, 41% of detected alterations in actionable genes were classified as actionable alterations. This is in contrast to the 2% of alterations that were classified as having no impact on function. Within this context, 44% of alterations in actionable genes were classified as variants of unknown significance. The remaining 13% were from one patient with 66 mutations in actionable genes. Given the hypermutated nature of this patient’s tumor, it was felt that none of the mutations would act as a driver or represented a good target for matched therapy; thus, they were not annotated (Fig 3A). Less than one half of actionable alterations were previously reported in the peer-reviewed literature. For the remainder, actionability was deduced from the type of mutation present.

Mutation burden varied substantially in the patient population. Not counting the sequencing results from the smaller panel, nearly 70% of tumor alterations were identified in patients with tumors that were found to be in the top quartile of overall tumor mutation burden. After the application of decision support, the number of actionable alterations was poorly correlated with mutation burden, as less than 50% of actionable alterations in actionable genes are in the top quartile of mutation load (Fig 3B). Thus, tumor mutation burden was not a good predictor of the presence of a clinically actionable alteration.

### Patient Outcomes

**FIG 4.**
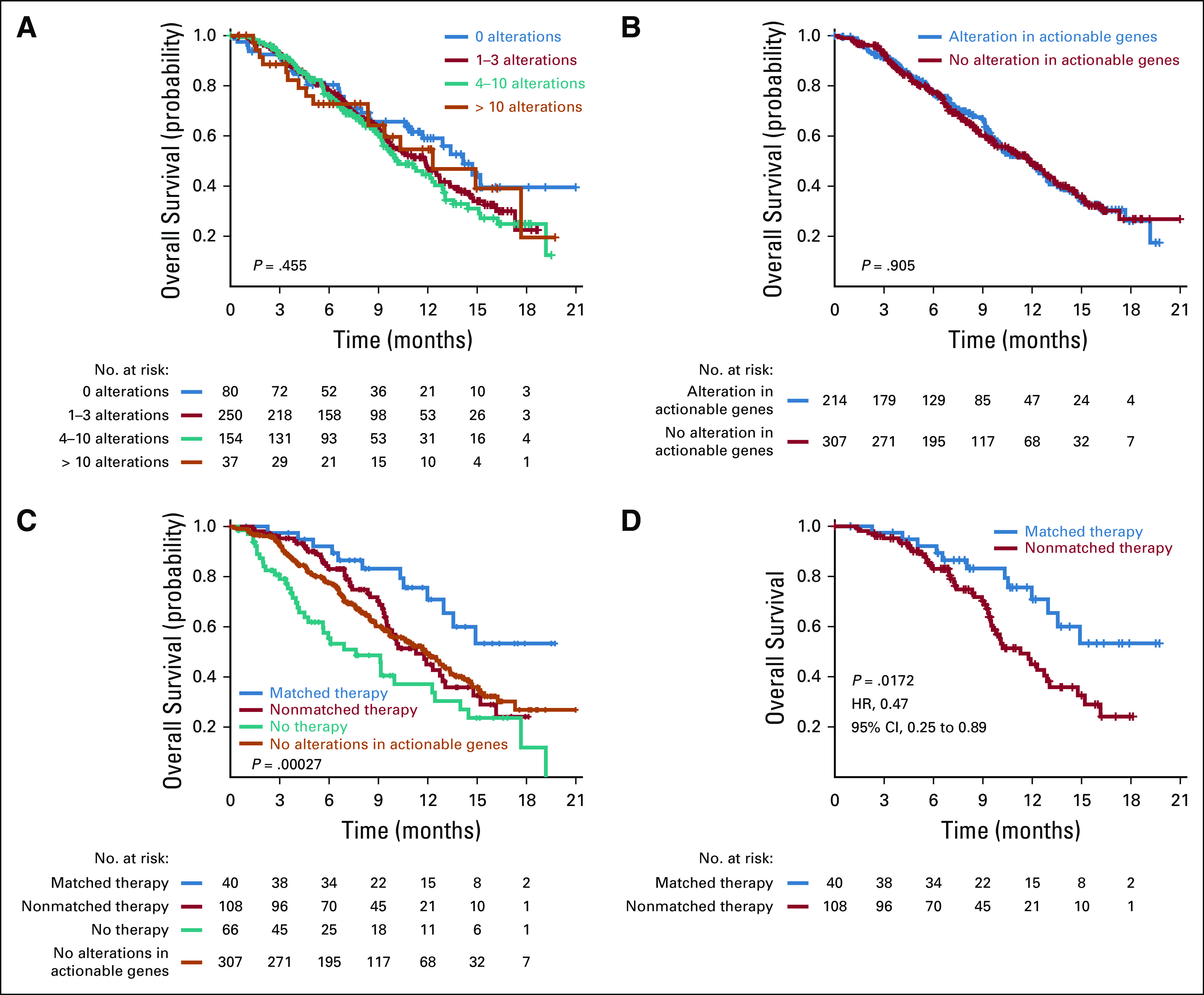
(A-D) Kaplan-Meier plots for overall survival by (A) total number of alterations (actionable and nonactionable) detected, (B) whether the patient had a tumor with an alteration in an actionable gene, (C) type of therapy and absence of alterations in actionable genes, and (D) whether the patient had a tumor with an alteration in an actionable gene on the 409-genes next-generation sequencing panel received matched treatment. Only treatment with matched targeted therapy (C and D) was associated with significantly improved survival (*P* = .017 compared with patients treated with nonmatched therapy). HR, hazard ratio.

**FIG 5.**
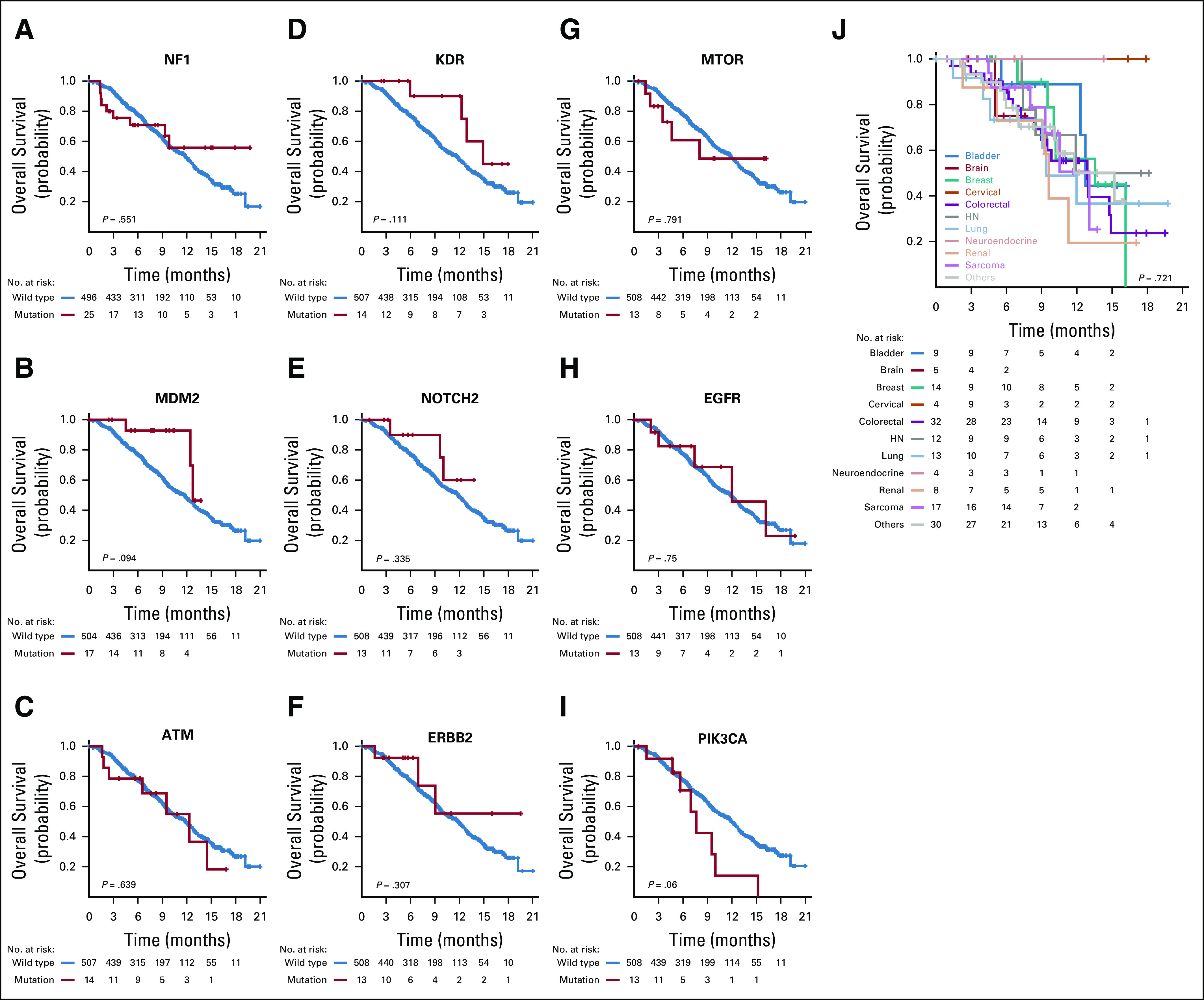
(A-J) Kaplan-Meier plots for overall survival for nine actionable genes commonly altered in clinical protocol patients (A-I) and for the most common cancer types represented in the protocol (J). No cancer type or alteration in a specific gene had a significant impact on patient survival. HN, head and neck.

Neither the number of overall gene alterations in the tumor, nor the presence of alterations in actionable genes significantly influenced patient survival (Figs 4A and 4B). Overall, most patients had poor survival (Fig 4C); however, patients with an alteration in an actionable gene who were treated with matched targeted therapy had significantly improved survival compared with the remainder of the cohort (Fig 4C) and patients who were treated with nonmatched therapy (Fig 4D; *P* = .017). Individual survival analyses among the most common tumor types represented in the protocol did not identify a specific cancer type that was significantly associated with improved survival (Fig 5). Among the nine genes that were commonly altered in protocol patients, none was associated with survival (Fig 5). Survival was assessed by manual curation of the medical record along with the date when a patient was last known to be alive as documented by a physician note or date of death provided by obituary, tumor registry, or reported by a family member. This study is limited by the lack of randomization; however, comparison of matched and unmatched patients helps to minimize concerns related to comorbidities or declining performance status as an explanation for the differences in outcomes observed in Figures 4C and 4D, as all patients in this analysis received another line of therapy, including participation in clinical trials.

Two patients were found to have deleterious germline mutations that were not previously known to the patient or oncologist—patient management was not altered in either case. One patient with optic glioma and a history of early-stage endometrial cancer had an *MSH6* germline mutation. A second patient with pleomorphic liposarcoma had a *TP53* germline mutation.

### Characteristics of Protocol Patients Compared With TCGA

Given that an eligibility criterion for enrollment was recurrence after frontline therapy, the molecular testing profiles for this patient cohort may differ from those previously published. TCGA did not select for patients with advanced cancers; therefore, we compared mutation frequencies in our cohort with those of the TCGA for colorectal adenocarcinoma, lung adenocarcinoma, and ovarian high-grade serous carcinoma.^19-21^ These three cancers were examined because of their relatively homogeneous histology and sufficient representation in the study. For colorectal cancer, *KRAS* mutation frequency was higher in our patient population, which was consistent with their resistance to epidermal growth factor receptor–directed therapy^22^ (Data Supplement). Protocol patients also had a significantly higher incidence of *TP53* mutations (72% *v* 52%; *P* = .004; data not shown). Only 3% of protocol colorectal cancers were microsatellite instability–high compared with 13% in TCGA. This is consistent with prior reports on the good prognosis of this subgroup.^23^ For ovarian high-grade serous carcinoma, alterations in actionable genes *CCNE1* (20%), *NF1* (12%), *PIK3CA* (18%), and *PTEN* (8%) were the most common in TCGA; however, only one protocol patient with ovarian cancer had a *CCNE1* amplification, whereas no alterations were observed in the other genes (Data Supplement). For lung adenocarcinoma, alterations in *EGFR*, *KRAS*, and *BRAF* were comparable between protocol patients and TCGA (data not shown). Only two clinically actionable genes—*CDKN2A* and *CDKN2B*—had lower frequencies of alteration in protocol patients with lung adenocarcinoma (Data Supplement).

## DISCUSSION

Survival for the overall cohort was poor in this institution-wide prospective protocol that enrolled patients with advanced solid tumor malignancies refractory to treatment. For 41% of these patients, we identified an alteration in a clinically actionable gene that was not detected by previous small-panel testing. With the aid of systematic decision support, this led to patients being directed to matched targeted therapy treatment regimens. Analysis of this enhanced gene set coupled with a targeted therapeutic approach was associated with improved survival. The overall utility of single—as opposed to sequential small-panel, then large-panel testing—large-panel NGS testing combined with decision support can be estimated by combining the 11% enrollment in clinical trials from our prior 46- and 50-gene panel study^18^ with the 8% derived from the incremental addition of the large-panel testing in this study. Although the result compares favorably with that from prior cohorts, such as MOSCATO (Molecular Screening for Cancer Treatment Optimization; 7%),^3^ ATTACC (Assessment of Targeted Therapies Against Colorectal Cancer; 20%),^24^ and SAFIR01 (High-Throughput Technologies to Drive Breast Cancer Patients to Specific Phase I/II Trials of Targeted Therapies; 13%),^25^ it highlights the fact that the matched targeted therapy approach still only benefits a small percentage of patients with cancer. Testing was performed on archival tissue rather than on a biopsy of the most recent metastatic site. As metastases can accumulate mutations over time, this represents another potential reason for limited patient benefit.

With increasing gene targets and matched drugs, it is becoming increasingly difficult for an individual physician to reasonably interpret the molecular findings from larger NGS panels. The importance of decision support in a comprehensive precision medicine strategy is highlighted by the fact that less than one half of actionable alterations in actionable genes had been reported previously in the literature. Over time, at least some of these variants of undetermined significance may be found to be activating mutations after they are examined in functional assays. Examples of variants that have been reclassified as activating mutations in a functional genomics platform^26^ include *PDGFRA* K385M, *PIK3CA* E110del, and *RET* D627N. Whereas decision support may be more readily available in academic practice, there are a number of publicly available and commercial databases that can provide such support. Publicly available options include My Cancer Genome, The Jackson Laboratory Clinical Knowledge Database, and OncoKB. Commercial providers include *N*-of-One, Molecular Health, and GenomOncology. The challenge to the community oncologist is deciphering which bioinformatics pipeline is most reliable. The reliability of quality decision support is crucial as patients with advanced cancers have a finite lifespan.

Patients with advanced cancers that are refractory to standard therapy may have mutational frequencies of individual genes that differ from published frequencies derived from unselected patients. This was apparent in patients with colorectal cancer and ovarian high-grade serous carcinoma in whom frequencies likely reflected a bias toward more clinically aggressive or treatment-resistant tumor biology. For example, frontline treatment of ovarian cancer is platinum-based chemotherapy; it is possible that, by selecting for patients with platinum-resistance, the protocol also selected for patients with ovarian cancers with therapy-driven alterations in the molecular landscape. The phenomenon is supported by the comparison of survival populations from stage-matched patients in TCGA and clinical trials, where patient survival from clinical trials was substantially lower.^27^ Patients with advanced cancers may have higher incidences of mutations associated with other forms of treatment resistance, such as *EGFR* T790M and *ESR1* mutations.^28^ The potentially unique molecular features in patients with advanced, chemotherapy-resistant cancers may represent one factor that contributes to the low success rate of the matched targeted therapy approach.

Treatment directed against actionable genes remains suboptimal. Pathways that were previously thought to be universally targetable have proven to be difficult to treat, such as the phosphatidylinositol 3-kinase/AKT pathway,^29^ or subject to variation in results by tumor type, such as *BRAF*^30^ or *HER2/3* mutations.^31,32^ In protocol patients, we were unable to ascertain any strong individual signals of activity for particular alterations in specific tumor types in part because of insufficient numbers of patients.

In conclusion, these results demonstrate the utility of large-panel NGS testing when combined with decision support. The derived benefit is realized in only a small subset of patients. Future efforts should emphasize high-quality and timely decision support and minimize barriers to patient enrollment, with the ultimate goal of broadly delivering precision medicine to patients with solid tumor malignancies. A critical step will involve identifying which combination of tumor type and alterations in actionable gene(s) is most efficacious when coupled with the most appropriate targeted therapeutics.
